# Pyruvate kinase M2 isoform deletion in cone photoreceptors results in age-related cone degeneration

**DOI:** 10.1038/s41419-018-0712-9

**Published:** 2018-07-03

**Authors:** Ammaji Rajala, Yuhong Wang, Krutik Soni, Raju V. S. Rajala

**Affiliations:** 1 Department of Ophthalmology, University of Oklahoma Health Sciences Center, Oklahoma City, OK USA; 2 Dean McGee Eye Institute, Oklahoma City, OK USA; 3 Department of Physiology, University of Oklahoma Health Sciences Center, Oklahoma City, OK USA; 4 Department of Cell Biology, University of Oklahoma Health Sciences Center, Oklahoma City, OK USA

## Abstract

The tumor form of pyruvate kinase M2 has been suggested to promote cellular anabolism by redirecting the metabolism to cause accumulation of glycolytic intermediates and increasing flux through the pentose phosphate pathway, which is a metabolic pathway parallel to glycolysis. Both rod and cone photoreceptors express the tumor form of pyruvate kinase M2. Recent studies from our laboratory show that PKM2 is functionally important for rod photoreceptor structure, function, and viability. However, the functional role of PKM2 in cones is not known. In this study, we conditionally deleted PKM2 in cones (cone-cre PKM2-KO) and found that loss of PKM2 results in the upregulation of PKM1 and a significant loss of cone function and cone degeneration in an age-dependent manner. Gene expression studies on cone-cre PKM2-KO show decreased expression of genes regulating glycolysis, PPP shunt, and fatty acid biosynthesis. Consistent with these observations, cones lacking PKM2 have significantly shorter cone outer segments than cones with PKM2. Our studies clearly suggest that PKM2 is essential for the anabolic process in cones to keep them alive for normal functioning and to support cone structure.

## Introduction

Vertebrate photoreceptors, like cancer cells and cells in other tissues that rely on active growth, use a specific isoform of pyruvate kinase, PKM2. PKM2 catalyzes the last step in glycolysis in the conversion of phosphoenolpyruvate (PEP) to pyruvate. Both rod and cone photoreceptor cells in the retina are highly metabolic, and a high level of anabolic activity is required for the outer segment renewal process^1^. In tumor cells, PKM2 is the predominantly expressed isoform, and investigators have suggested that PKM2 promotes the accumulation of glycolytic intermediates and increases flux through the pentose phosphate pathway (PPP) for anabolic processes, mainly to synthesize lipids, proteins, and ribonucleic acids^2^. The NADPH generated through PPP is also essential for antioxidant metabolism^3^.

Our recent studies suggest that both the metabolic and transcriptional regulatory functions of PKM2 influence rod photoreceptor structure, function, and viability^4^. Interestingly, when we deleted PKM2 in rods, we found that building up glycolytic intermediates and increasing NADPH levels are not sufficient enough to increase anabolic activity and cell survival^4^. In PKM2 knockout rods, the PKM1 expression is upregulated, yet it did not catalyze the PEP to pyruvate; as a result, we observed increased glycolytic intermediates^4^. These findings indicate the influence that regulating the final step in glycolysis can have on cells that rely on anabolic activity. Our recent study indicated that there are other important reasons why PKM2 is so highly expressed in cells requiring highly active anabolic activity^4^. It is well known that mutations in rods cause the secondary death of cones in a majority of retinal degenerative diseases. Interestingly, deletion of PKM2 in rods had no effect on cone structure and function^4^. PKM2 is also expressed in cones; however, its functional role in cones is unknown. In the present study, we conditionally deleted PKM2 in cones and examined the functional role of PKM2 on cone and rod structure and function.

## Results

### Expression of PKM2 in cone photoreceptor cells

**Fig. 1:**
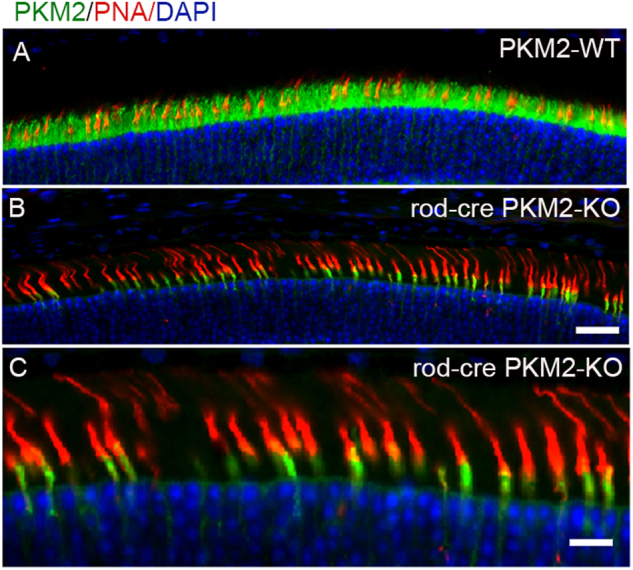
Immunofluorescence analysis of PKM2 in wild-type and rod-cre PKM2-KO mice. Prefer-fixed sections of PKM2-WT (**a**) and rod-cre PKM2-KO (**b**) mouse retinas were subjected to immunofluorescence with anti-PKM2 (green) and anti-PNA (red) antibodies. Panel (**c**) is an enlarged image of (**b**) showing PKM2 expression in cones. Scale bar = 50 μm (**a**, **b**) and 20 μm for panel (**c**)

To demonstrate the expression of PKM2 in wild-type mouse cones, we examined the expression of PKM2 in mouse retinas lacking PKM2 in rod photoreceptor cells (rod-cre PKM2-KO). PKM2-wild-type (PKM2-WT) and rod-cre PKM2-KO mouse retinas were immunostained with PKM2 and peanut agglutinin (PNA). The results indicated that PKM2 is expressed in rod photoreceptors of PKM2-WT mice and co-localized with PNA, suggesting the possible expression of PKM2 in cones (Fig. 1a). In rod-cre PKM2-KO mouse retinas, the PKM2 was absent from rods and we observed clear PKM2 immunofluorescence in cones co-localized with PNA (Fig. 1b). An enlarged view of the co-localization of PKM2 and PNA showed some expression of PKM2 in the outer segments of cones (yellow signal) as PNA labels the cone outer segment sheets (Fig. 1c). These observations suggest the expression of PKM2 in mouse cones.

### Cone photoreceptor cell survival in mice lacking PKM2 in rods

To determine whether loss of PKM2 in rods affects cone survival, we stained 20-week-old PKM2-WT and rod-cre PKM2-KO mouse retinal sections with cone photoreceptor markers, PNA, M-opsin, and S-opsin. The results indicate that there was no difference in the expression of M-opsin and S-opsin between PKM2-WT and rod-cre PKM2-KO mouse retinas (Fig. S1). The PNA staining is indistinguishable between PKM2-WT and -rod-cre PKM2-KO mouse retina indicates that cone density was well preserved (data not shown), suggesting that loss of PKM2 in rods has no effect on cone survival.

### Conditional deletion of PKM2 in cone photoreceptor cells

**Fig. 2:**
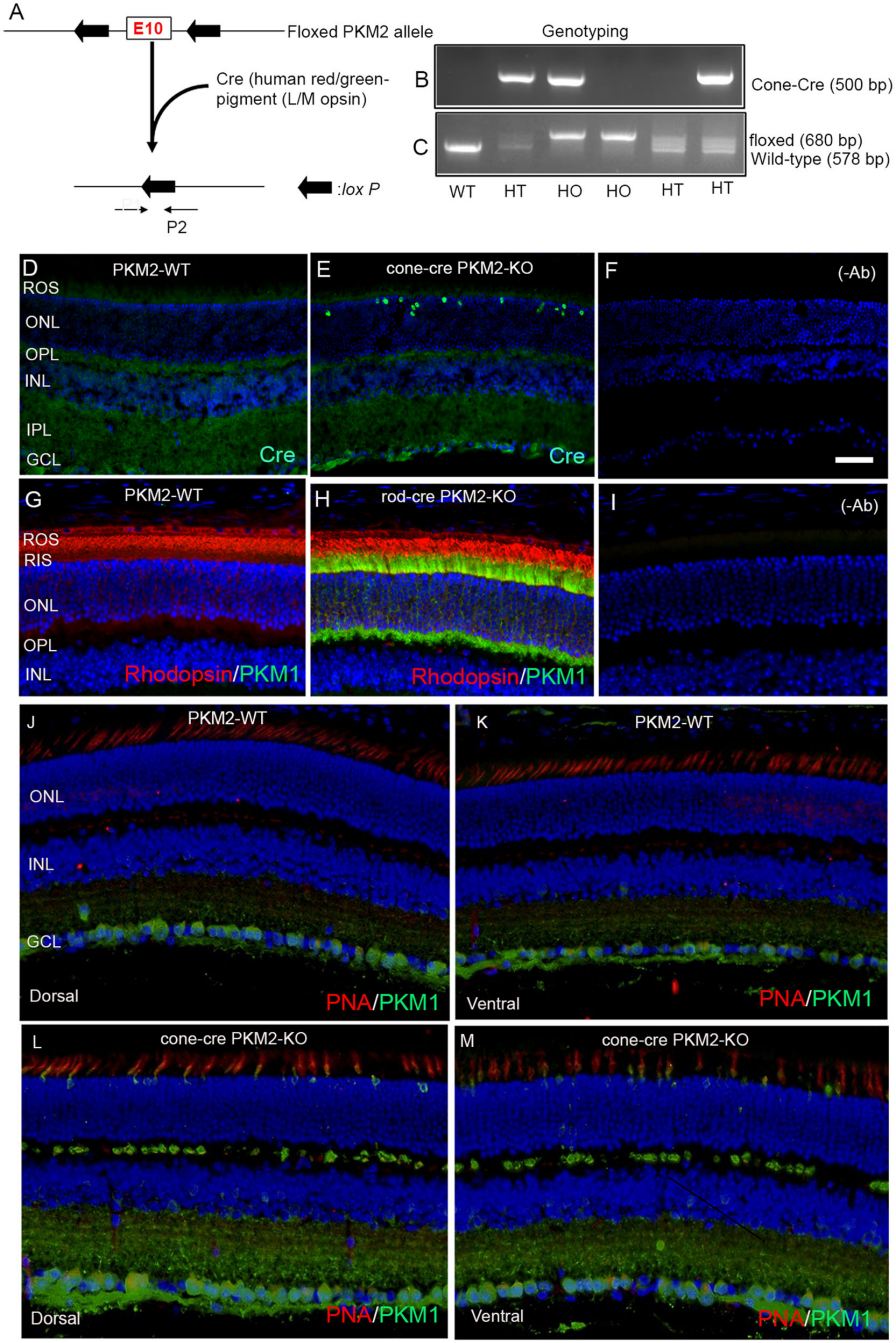
Generation of cone-conditional PKM2-KO mice. Schematic diagram of *loxP* floxed PKM2 loci. Cone photoreceptor-specific PKM2 KO mice were generated by breeding mice with an exon 10 floxed *PKM* gene with mice that express Cre recombinase under the control of human red/green pigment promoter (**a**). Primer pairs P1 and P2 were used to identify the wild-type, cone-Cre, and the floxed PKM2 alleles (**b**, **c**). Cryosections were prepared from PKM2-WT and cone-cre-PKM2-KO mouse retinas and were immunostained with an anti-Cre antibody (**d**, **e**). Prefer-fixed sections of 8-week-old PKM2-WT (**g**) and rod-cre PKM2-KO (**h**) mouse retinas were subjected to immunofluorescence with anti-rhodopsin (red) and PKM1 (green) antibodies. Prefer-fixed sections of 12-week-old PKM2-WT (**j**, **k**) and cone-cre PKM2-KO (**l**, **m**) mouse retains were stained with PNA (red) and PKM1 (green) antibodies, and images were captured on both dorsal and ventral regions of the retina. Panels (**f**) and (**i**) represent the omission of primiary antibody. ROS rod outer segments, RIS rod inner segments, ONL outer nuclear layer, OPL outer plexiform layer, INL inner nuclear layer, IPL inner plexiform layer, GCL ganglion cell layer. Scale bar = 50 μm

To determine the functional role of PKM2 in cones, we mated mice in which PKM2 exon-10 was floxed with mice carrying Cre-recombinase under the control of human red/green pigment promoter^5^. Cre-mediated excision of floxed exon-10 in the *PKM* gene results in the deletion of PKM2 in cones (Fig. 2a), and the genotypes were identified by genomic PCR (Fig. 2b, c). Immunohistochemistry with Cre antibody shows the expression of Cre-recombinase in cone photoreceptor nuclei in cone-cre PKM2-KO mouse retinas and Cre expression are absent from PKM2-WT mouse retinas (Fig. 2d-f ). The proper targeting of Cre protein expression in cones is an indirect way of confirming the gene deletion when it encounters a floxed allele.

### Confirmation of PKM2 deletion in cone photoreceptor cells

The PKM1 and PKM2 isoforms arise from the *PKM* gene via alternative transcript splicing, such that exon 9 is exclusive to PKM1 and exon 10 is exclusive to PKM2^6^. The PKM2 expression can be favored over PKM1 via suppression of exon 9 inclusion by the splicing repressors^7^. These observations suggest that either PKM2 or PKM1 be expressed, but not both isoforms, in a given cell type. We and others have shown that deletion of PKM2 in rods results in the upregulation of PKM1 in photoreceptor cells^4,8,9^. We used this finding to confirm the deletion of PKM2 and stained PKM2-WT and cone conditional PKM2-KO (cone-cre PKM2-KO) mouse retinas with PKM1 and PNA to examine the upregulation of PKM1 in cones. For control experiments, PKM2-WT and rod-cre PKM2-KO mouse retina sections were immunostained with PKM1 and rhodopsin antibodies. The results indicate that PKM1 expression is absent from PKM2-WT mouse retina and is predominantly expressed in rod-cre PKM2-KO mouse retina, but the rhodopsin expression remains same between PKM2-WT and rod-cre PKM2-KO mouse retinas (Fig. 2g-i). Similarly, our results also indicate that PKM1 expression is absent from PKM2-WT mouse cones in both dorsal and ventral regions (Fig. 2j, k), but PKM1 expression is clearly visualized in cone-cre PKM2-KO mouse cones in both dorsal and ventral regions (Fig. 2l, m). These experiments confirm the deletion of PKM2 in cones of cone-cre PKM2-KO mice.

**Fig. 3:**
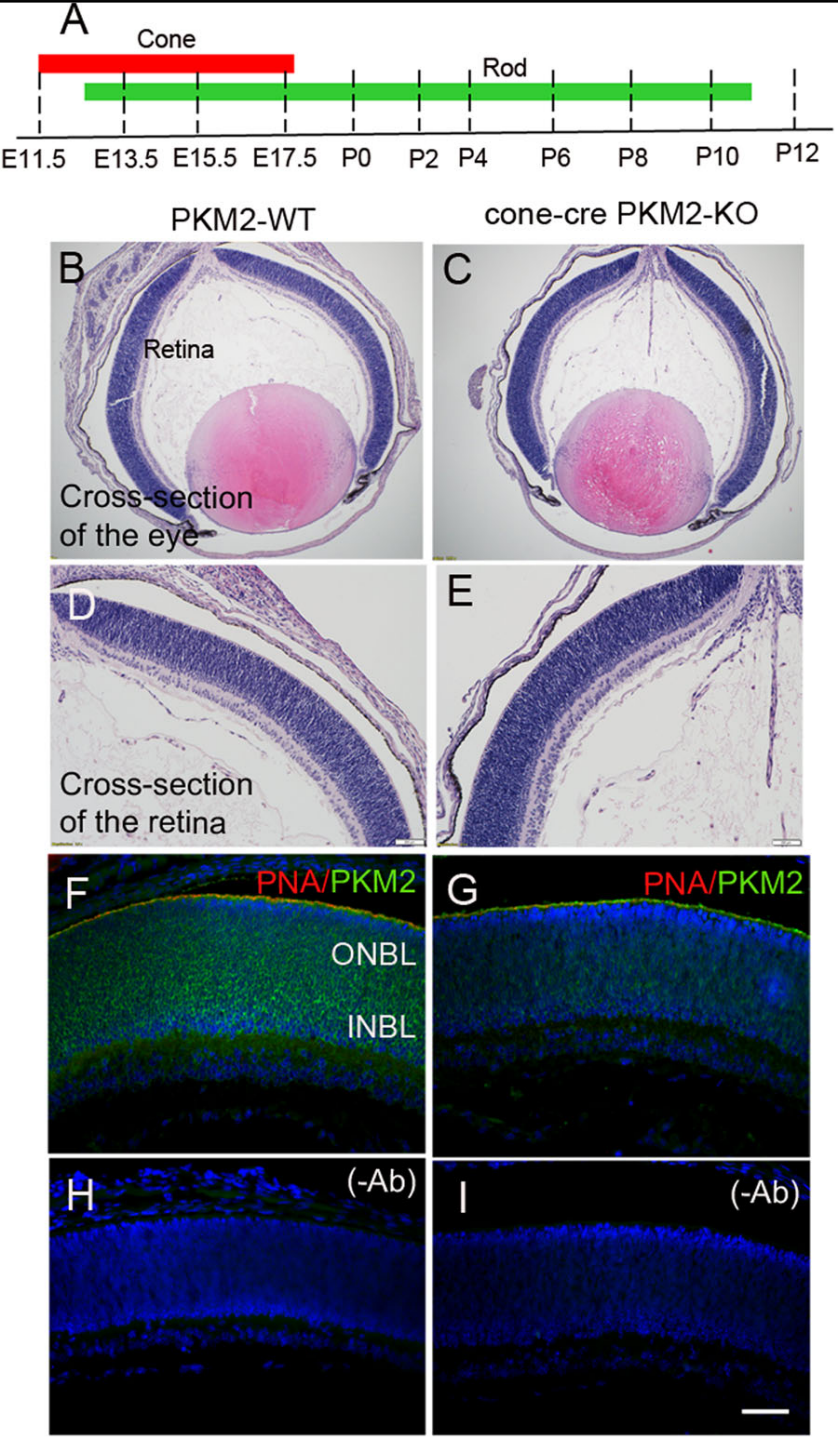
Expression of PKM2 in the developmental retina. Developmental scheme of cone and rod photoreceptors showing that cone development precedes rod development (**a**). After euthanization, whole P0 mouse heads were harvested, cut in half with one eye on each side, and were subjected to prefer-fixation followed by paraffin embedding. Genotyping was performed on mouse tail DNA to distinguish cone-cre PKM2-KO mice from PKM2-WT mice, as described in Methods. Sections were prepared from PKM2-WT (**b**, **d**) and cone-cre-PKM2-KO (**c**, **e**) mice and stained with hematoxylin and eosin. Panels (**b**) and (**c**) show the cross-section of the eye. Panels (**d**) and (**e**) show the cross-section of the retina. Immunohistochemistry was performed on PKM2-WT (**f**) and cone-cre PKM2-KO (**g**) retina sections with PNA (red) and PKM2 (green). Panels (**h**) and (**i**) represent the omission of primary antibodies. ONBL outer neuroblastic layer, INBL inner neuroblastic layer. Scale bar = 50 μm

In retinal development, cone development starts around embryonic day (E) 11.5 and completes by E17.5, whereas rod development starts around E12 and completes around postnatal day (P) 10 (Fig. 3a). Retinal sections were cut from whole P0 mouse heads (Fig. 3b-e), and we performed immunohistochemistry using PKM2 and PNA antibodies. The results indicate that PKM2 expression was detected at P0 in the outer (ONBL) and inner neuroblastic (INBL) layers, whereas PNA staining was observed on the border of the ONBL in PKM2-WT retina (Fig. 3f). In cone-cre PKM2-KO P0 retina, the expression of PKM2 was reduced in INBL and considerably more reduced in ONBL. However, we found no change in PNA staining pattern on the border of the ONBL (Fig. 3g and Fig. S2A and B). We also stained P0 sections with Cre and M-opsin antibodies, and the results indicate the presence of Cre immunoreactivity in cone-cre PKM2-KO mouse retina and absence in PKM2-WT mouse retina (Fig. S2A and B). The M-opsin expression is indistinguishable between PKM2-WT and cone-cre PKM2-KO mouse retina (Fig. S2C and D). Rhodopsin expression was absent in P0 retinas (Fig. S2I and J) and can be visualized in P7 retinas (Fig. S2K). A lot of cone markers come on even before birth and we could see a reduction of PKM2 in P0 cone-cre PKM2-KO mouse retina compared to P0 PKM2-WT retina (Fig. 3f, g). Consistent with this observation, we could also see the expression of M-opsin in both PKM2-WT and cone-cre PKM2-KO retinas and the expression of Cre as well in P0 retinas of cone-cre PKM2-KO mice (Fig. S2B). Collectively, our data suggest the loss of PKM2 in the cones of cone-cre PKM2-KO mice.

### Functional and structural characterization of cone-cre PKM2-KO mice

**Fig. 4:**
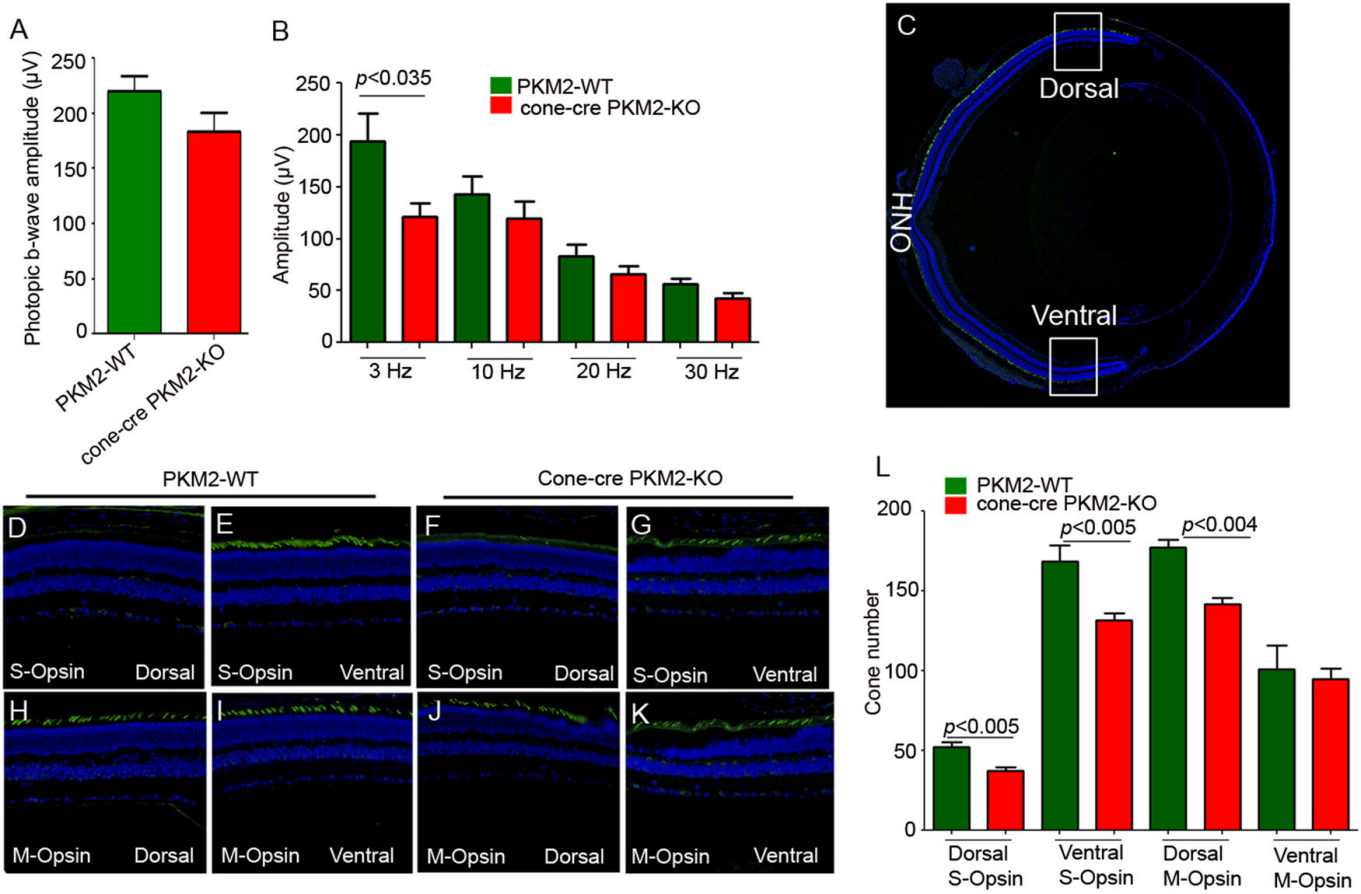
The function and structure of the 12-week-old cone-cre PKM2-KO mouse retina. Cone function (photopic b-wave) and cone flicker electroretinographic analysis were performed on 12-week-old PKM2-WT and cone-cre PKM2-KO mice. Photopic b-wave amplitudes were performed at a flash intensity of 3.3 log cd s/m^2^ (**a**). Isolated cone photoreceptor components of the photopic flicker ERG were done at 3, 10, 20, and 30 Hz (**b**). Panel (**c**) represents staining of the retina with M-opsin (green) and nuclei (blue) on a section of PKM2-WT retina at 12 weeks (Scale bar = 100 μm). Higher expression of M-opsin dorsally than ventrally. Panels (**d**–**k**) represent the difference of expression of S-opsin (S-opsin positive cones) and M-opsin (M-opsin positive cones) in dorsal and ventral regions of the retina from 12-week-old PKM2-WT and cone-cre PKM2-KO mice. Panel (**l**) represents the quantification of the number of cones in dorsal and ventral regions of the retina counted starting from the optic nerve head (ONH). Data are mean ± SEM (*n* *=* *6*). Significance, if any, is indicated on each panel. The images shown are representative of six retinas examined from PKM2-WT and cone-cre PKM2-KO mice

We found a decreased cone function in 12 weeks old cone-cre PKM2-KO mice compared to PKM2-WT mice. However, this difference was not statistically significant (Fig. 4a). Flicker ERG is a type of full-field ERG (ffERG) in which light flashes quickly (3–30 Hz) and the rod photoreceptor cannot follow such a fast stimulus^10^. Cone flicker ERG at 3, 10, 20, and 30 Hz flickering stimuli showed significantly reduced amplitudes at 3 Hz flickering stimuli in cone-cre PKM2-KO mice compared with amplitudes in PKM2-WT mice (Fig. 4b). No significant differences were observed at higher flickering stimuli between PKM2-WT and cone-cre PKM2-KO mice. To determine whether the decreased cone function in cone-cre PKM2-KO mice is due to a loss of cone photoreceptor cells, we examined S-opsin positive cones and M-opsin positive cones in dorsal and ventral regions of retina by immunohistochemistry (Fig. 4c-k) and quantified the number of cones in dorsal and ventral regions of the retina starting from the optic nerve head (Fig. 4l). S-opsin positive cones are predominant in the ventral region of the retina compared to the dorsal region, whereas M-opsin positive cones are distributed in both dorsal and ventral regions but have slightly higher distribution in the dorsal region of the retina^11^. These results indicate a significantly reduced number of S-opsin positive cones in both dorsal and ventral regions and a significantly reduced number of M-opsin positive cones in the dorsal region of the retina in cone-cre PKM2-KO mice compared to PKM2-WT mice (Fig. 4l). We found no change in the number of M-opsin positive cones in the ventral region between PKM2-WT and cone-cre PKM2-KO mice (Fig. 4l). These observations suggest that, even though there was no significant change in the cone function in cone-cre-PKM2 KO at 12 weeks compared to PKM2-WT, we could observe the loss of both S-opsin and M-opsin positive cones in cone-cre PKM2-KO mice.

**Fig. 5:**
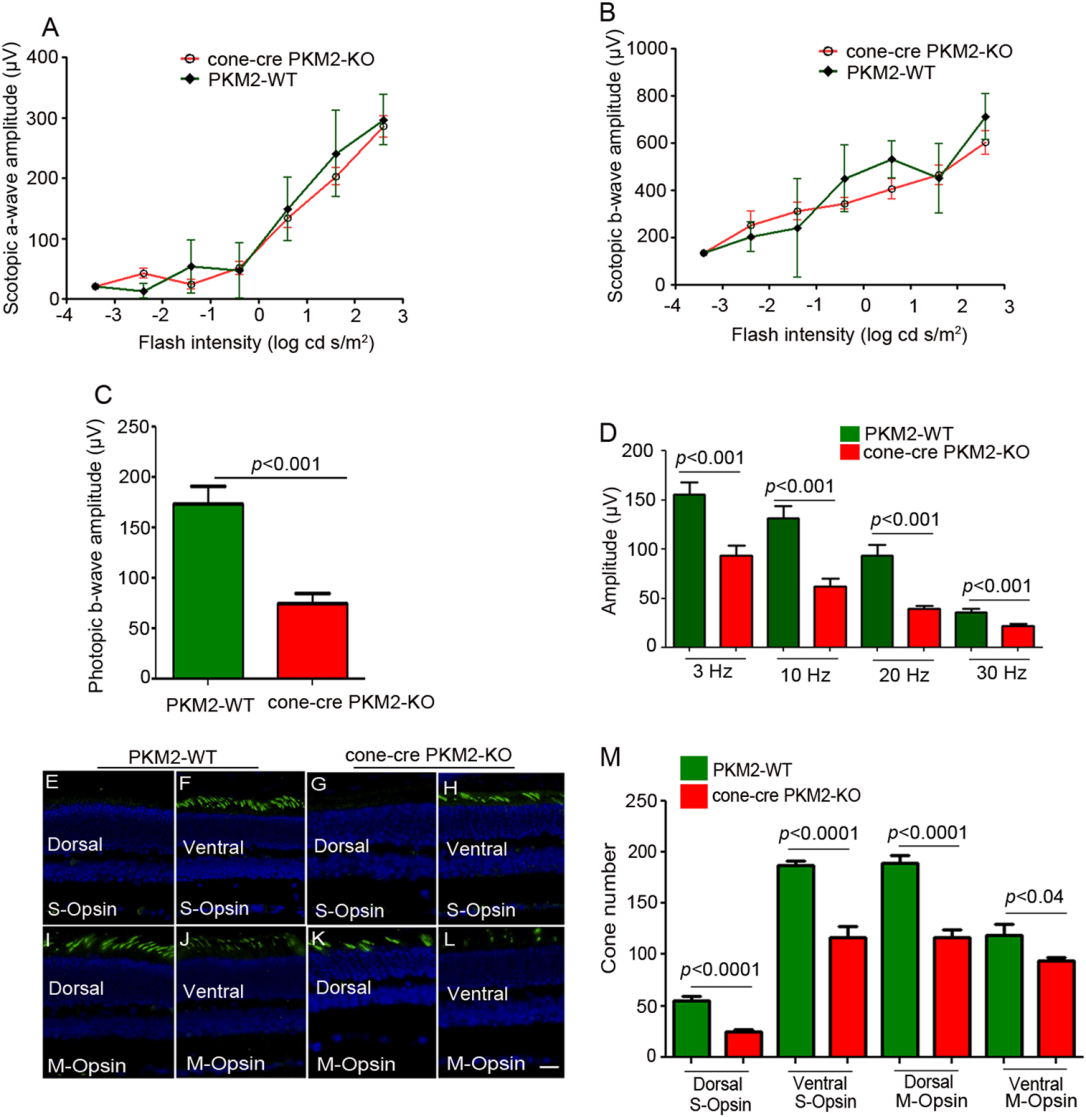
The function and structure of the 28-week-old cone-cre PKM2-KO mouse retina. Scotopic a-wave (**a**), scotopic b-wave (**b**), photopic b-wave (**c**), and cone flicker (**d**) electroretinographic analysis was performed on 28-week-old PKM2-WT and cone-cre PKM2-KO mice. Scotopic a-wave and scotopic b-wave amplitudes were carried out at different flash intensities (−3.4, −2.4, −1.4, −0.4, 0.6, 1.6, and 2.6 log cd s/m^2^), whereas photopic b-wave amplitudes were performed at a flash intensity of 3.3 log cd s/m^2^. Isolated cone photoreceptor components of the photopic flicker ERG were done at 3, 10, 20, and 30 Hz. Data are mean ± SEM (*n* = *13*). Significance, if any, is indicated on each panel. Panels (**e**–**l**) represent the difference of expression of S-opsin (S-opsin positive cones) and M-opsin (M-opsin positive cones) in dorsal and ventral regions of the retina from 28-week-old PKM2-WT and cone-cre PKM2-KO mice. Panel (**m**) represents the quantification of the number of cones in dorsal and ventral regions of the retina counted starting from the optic nerve head (ONH). Data are mean ± SEM (*n* = *8*). Significance, if any, is indicated on each panel. Scale bar = 50 μm. The images shown are representative of six retinas examined from PKM2-WT and cone-cre PKM2-KO mice

We followed up with ERG when the mice were 28 weeks old and measured scotopic a-wave, scotopic b-wave, photopic b-wave amplitudes, and cone flicker ERG at different flash intensities. The results showed no significant difference in the scotopic a-wave (Fig. 5a) and scotopic b-wave (Fig. 5b) amplitudes between PKM2-WT and cone-cre PKM2-KO mice at each measured flash intensity. The photopic b-wave amplitudes (Fig. 5c) and cone flicker ERG (Fig. 5d) of cone-cre PKM2-KO mice were significantly lower than those of PKM2-WT mice, suggesting that loss of PKM2 in cones affects cone function in an age-dependent manner. Collectively, these experiments suggest that loss of PKM2 in cones affects cone function. To determine the effect of loss of PKM2 on cone structure, we carried out immunohistochemistry with S-opsin and M-opsin (Fig. 5e-l) and quantified the S-opsin and M-opsin positive cones in dorsal and ventral regions of the retina (Fig. 5m). The results indicate a significantly reduced number of S-opsin positive and M-opsin positive cones in both dorsal and ventral regions of the retina in cone-cre PKM2-KO mice compared to PKM2-WT mice (Fig. 5m). These observations suggest that loss of cone function in cone-cre PKM2-KO mice at 28 weeks is correlated with the loss of S-opsin and M-opsin positive cones in cone-cre-PKM2 KO mice.

**Fig. 6:**
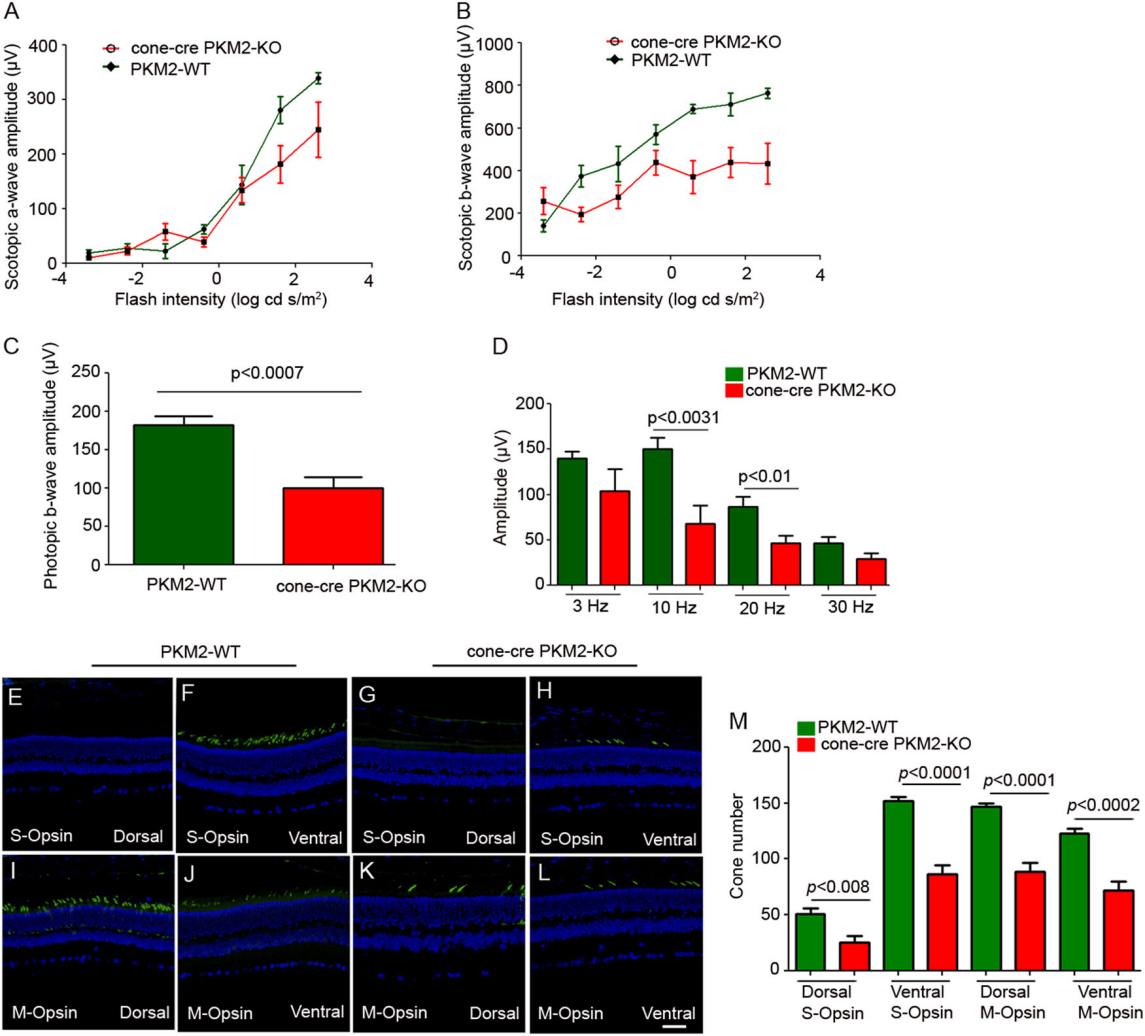
The function and structure of the 56-week-old cone-cre PKM2-KO mouse retina. Scotopic a-wave (**a**), scotopic b-wave (**b**), photopic b-wave (**c**), and cone flicker (**d**) electroretinographic analysis was performed on 56-week-old PKM2-WT and cone-cre PKM2-KO mice. Scotopic a-wave and scotopic b-wave amplitudes were carried out at different flash intensities (−3.4, −2.4, −1.4, −0.4, 0.6, 1.6, and 2.6 log cd s/m^2^), whereas photopic b-wave amplitudes were performed at a flash intensity of 3.3 log cd s/m^2^. Isolated cone photoreceptor components of the photopic flicker ERG were done at 3, 10, 20, and 30 Hz. Data are mean ± SEM (*n* *=* *13*). Significance, if any, is indicated on each panel. Panels (**e**–**l**) represent the difference of expression of S-opsin (S-opsin positive cones) and M-opsin (M-opsin positive cones) in dorsal and ventral regions of the retina from 56-week-old PKM2-WT and cone-cre PKM2-KO mice. Panel (**m**) represents the quantification of the number of cones in dorsal and ventral regions of the retina counted starting from the optic nerve head (ONH). Data are mean ± SEM (*n* = 8). Significance, if any, is indicated on each panel. Scale bar = 50 μm. The images shown are representative of six retinas examined from PKM2-WT and cone-cre PKM2-KO mice

To determine the effect of loss of PKM2 in cones on aged mice, we examined cone function and structure in 56-week-old PKM2-WT and cone-cre PKM2-KO mice. The results indicate that there was no significant difference in scotopic a-wave amplitudes between PKM2-WT and cone-cre PKM2-KO mice (Fig. 6a), but there was a significant decrease of scotopic b-wave amplitudes (Fig. 6b) and photopic b-wave amplitudes (Fig. 6c) in cone-cre PKM2-KO mice compared with PKM2-WT mice. Cone flicker ERG at 3, 10, 20, and 30 Hz flickering stimuli show reduced amplitudes in cone-cre PKM2-KO mice compared with PKM2-WT mice, but a significant decrease of cone flicker was observed at 10 and 20 Hz in cone-cre PKM2-KO mice compared with PKM2-WT mice (Fig. 6d). We also found significantly reduced number of S-opsin and M-opsin positive cones in both dorsal and ventral regions of the retina in cone-cre PKM2-KO mice compared to PKM2-WT mice (Fig. 6e-m). These observations suggest that loss of cone function in cone-cre PKM2-KO mice at 56 weeks is correlated with the loss of S-opsin and M-opsin positive cones in cone-cre-PKM2 KO mice. Collectively, our data suggest an age-dependent loss of S-opsin and M-opsin positive cones in both dorsal and ventral regions of the retina in cone-cre-PKM2 KO mice compared to PKM2-WT mice (Fig. S3).

**Fig. 7:**
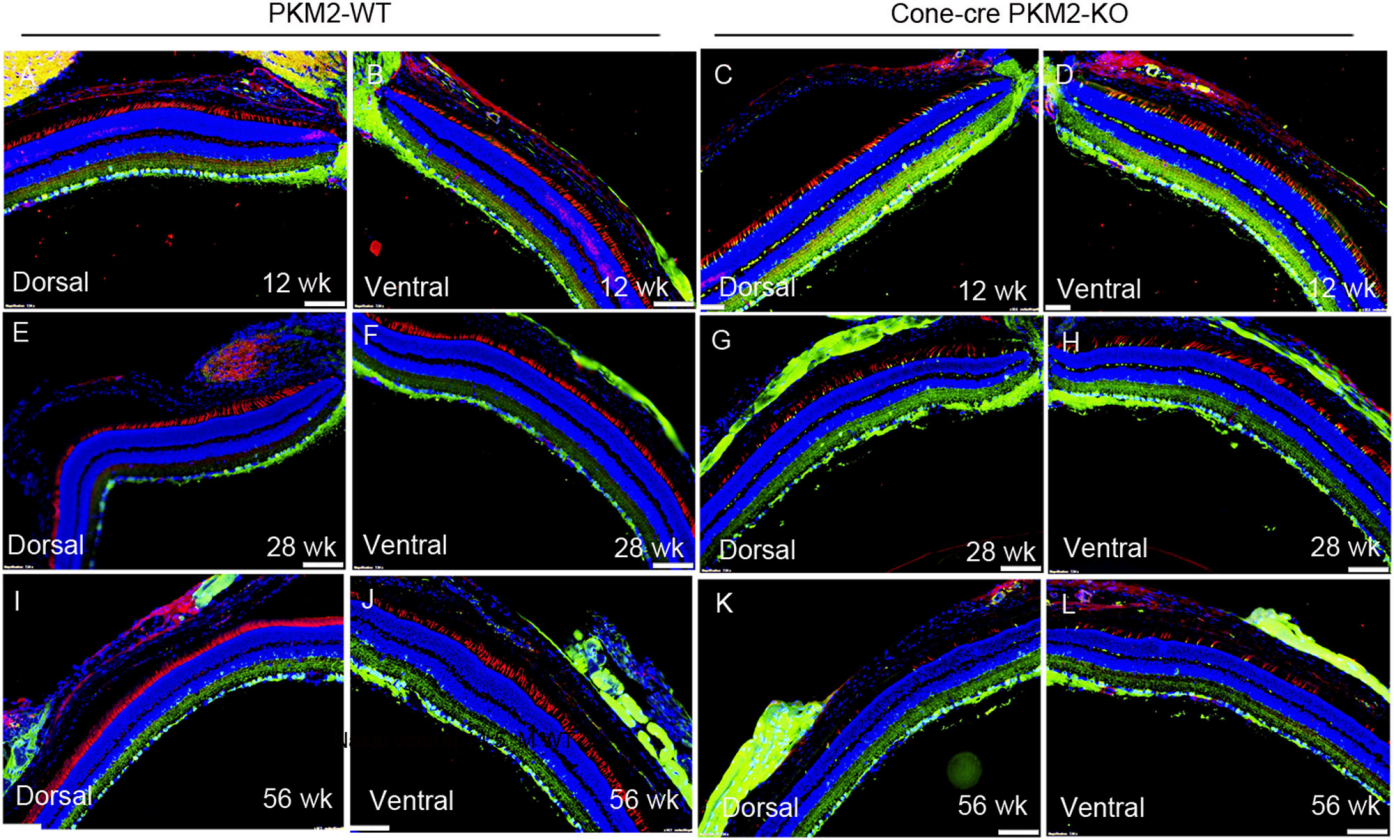
Cone photoreceptor integrity in cone-cre PKM2-KO mice. Prefer-fixed sections of 12-week-old, 28-week-old, and 56-week-old PKM2-WT (**a**, **b**, **e**, **f**, **i**, **j**) and cone-cre PKM2-KO (**c**, **d**, **g**, **h**, **k**, **l**) mouse retinas were subjected to immunofluorescence with anti-PNA (red) and anti-PKM1 (green) antibodies. Images were captured from dorsal and ventral regions of the retina. The images shown are representative of five retinas examined from PKM2-WT and cone-cre PKM2-KO mice. Scale bar = 100 μm

To corroborate our findings, we stained the retinal sections of PKM2-WT and cone-cre PKM2-KO mice with PNA and PKM1 in both dorsal and ventral regions of the retina. The results indicate loss of PNA positive cones in cone-cre PKM2-KO mouse retina sections in both dorsal and ventral regions in an age-dependent manner compared to PKM2-WT mouse retina sections, whereas PKM1 expression could be seen only in cone-cre PKM2-KO mouse retinas but not in PKM2-WT retinas (Fig. 7). We also found that loss of PKM2 has no effect on rod structure when we examined the retinal morphology of 28-week-old cone-cre PKM2-KO mouse retinal sections with hematoxylin and eosin staining (H & E). We found no difference in the structure or indication of rod photoreceptor degeneration in cone-cre PKM2-KO mouse retinas, and the retina structure was comparable to that of PKM2-WT mouse retina (Fig. S4).

**Fig. 8:**
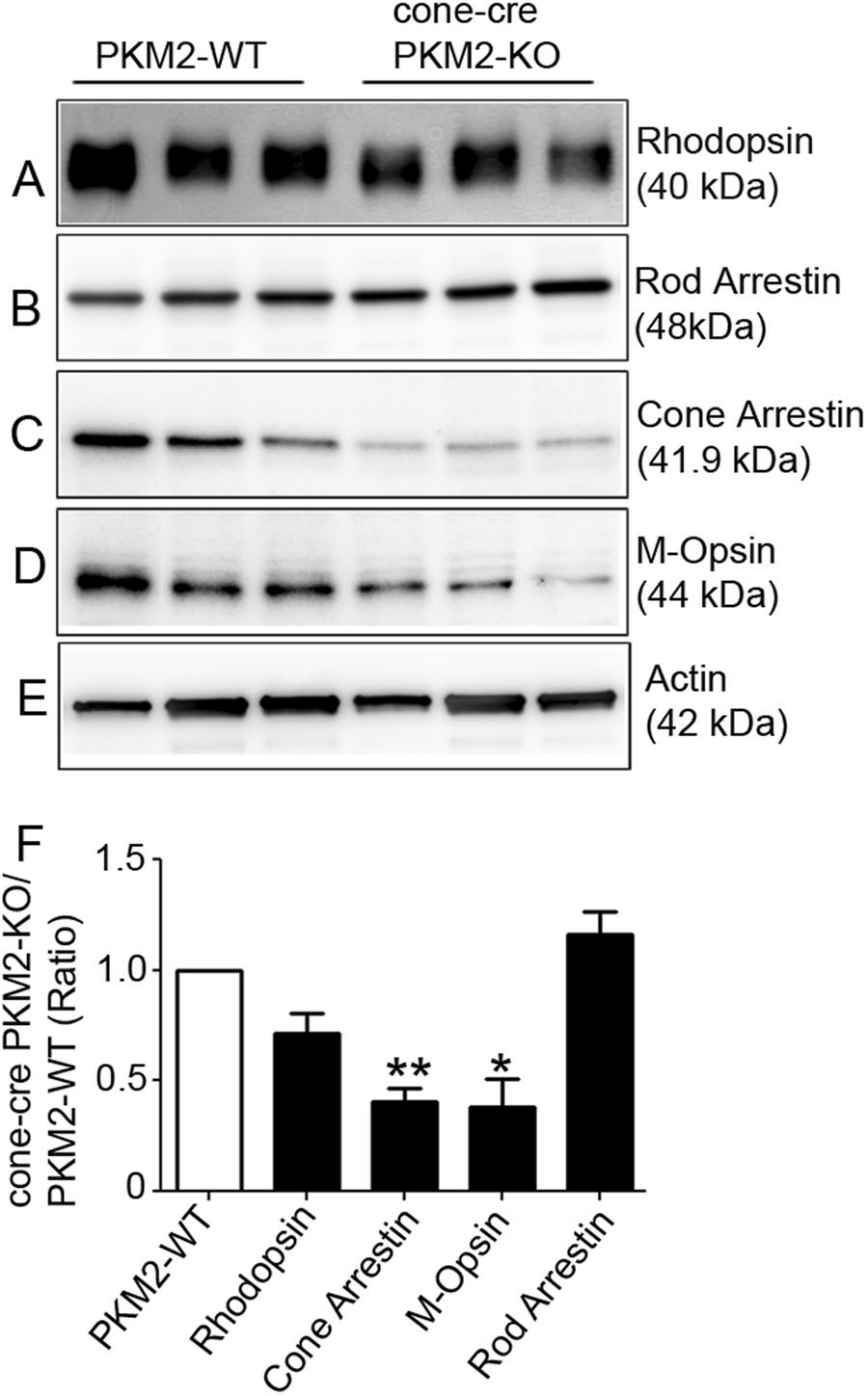
Expression levels of PKM2 and photoreceptor-specific proteins in PKM2-WT and cone-cre PKM2-KO mice. Retinal homogenates (5.0 µg protein) from three independent PKM2-WT and cone-cre PKM2-KO mice were subjected to immunoblot analysis with anti-rhodopsin (**a**), anti-rod arrestin (**b**), anti-cone arrestin (**c**), anti-M-opsin (**d**), and anti-actin (**e**) antibodies. We normalized the protein expression to rod arrestin (**f**) and then calculated the ratios (cone-cre PKM2-KO/PKM2-WT). Data are mean ± SEM (*n* = *3*). **p < 0.01, *p < 0.03. The molecular weight of each protein is indicated in the parentheses

To corroborate our immunohistochemistry results, we carried out immunoblot analysis on PKM2-WT and cone-cre PKM2-KO mouse retinal lysates with rhodopsin, rod arrestin, cone arrestin, M-opsin, and actin antibodies (Fig. 8a-e), and normalized the protein levels to rod arrestin (Fig. 8f). The results indicate significantly decreased levels of cone markers, cone arrestin, and M-opsin in cone-cre PKM2-KO mouse retinas compared with PKM2-WT mouse retinas (Fig. 8). There were no significant differences in the levels of rhodopsin or rod arrestin between cone-cre PKM2-KO and PKM2-WT retinas (Fig. 8). These experiments suggest that a loss of PKM2 in cones causes progressive cone degeneration.

### Effect of loss of PKM2 in cones on genes involved in energy metabolism, lipid biosynthesis, and cone photoreceptor maintenance

**Fig. 9:**
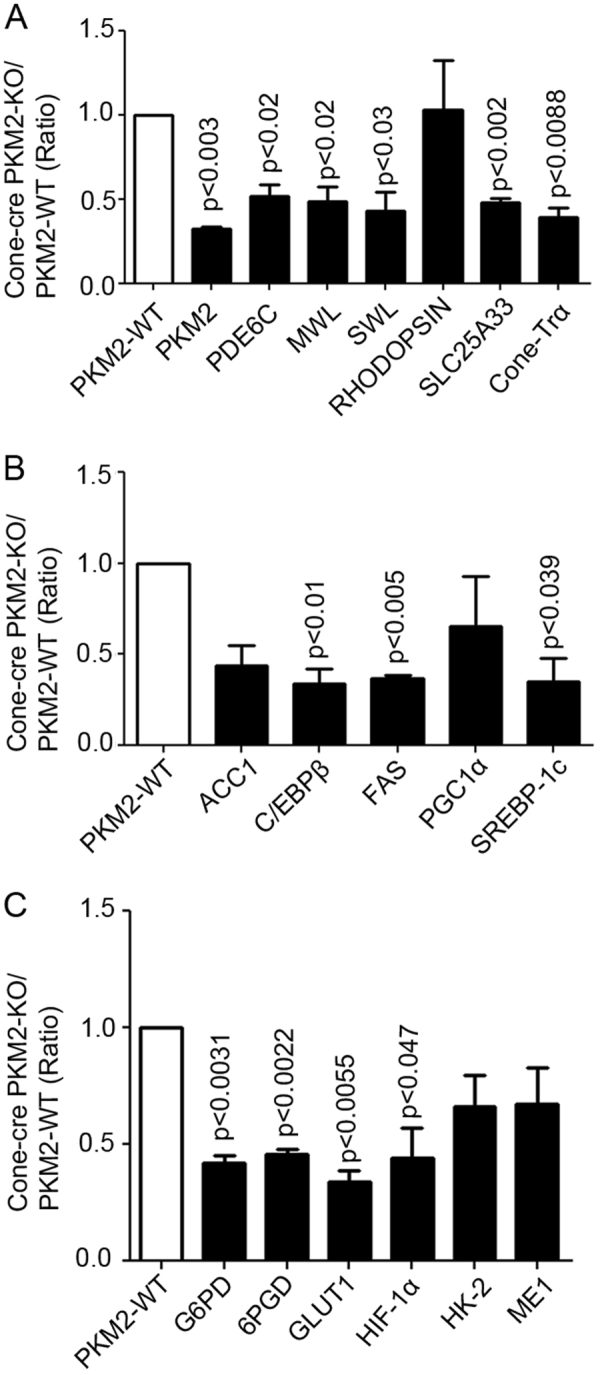
Expression of the photoreceptor, fatty acid biosynthetic, glycolytic, and pentose phosphate pathway-regulated gene expression in cone-cre PKM2-KO mice. Equal amounts of retinal total RNA reverse transcribed to cDNA from three independent PKM2-WT and cone-cre PKM2-KO mice were used for real-time (RT)-PCR and were normalized to 18 S rRNA levels and ratios were calculated (cone-cre PKM2-KO/PKM2-WT). Data are mean ± SEM (*n* *=* *3*). Significance, if any, is indicated on each panel. Panel (**a**) represents the expression of rod photoreceptor, cone photoreceptor, and mitochondrial genes in cone-cre PKM2 KO mice. Panel (**b**) represents the expression of fatty acid biosynthetic genes, whereas panel (**c**) represents the expression of pentose phosphate pathway-regulated genes in cone-cre PKM2 KO mice. MWL medium wavelength opsin, SWL short wavelength opsin

To determine the loss of PKM2 on cone photoreceptor gene expression, RNA was isolated from 3-month-old PKM2-WT and cone-cre PKM2-KO mice. Then, it was reverse transcribed to cDNA and subjected to real-time PCR using the primers listed in Table 1. The examined genes were quantified by normalizing to 18S ribosomal RNA. It is interesting to note that PKM2 is significantly down-regulated in cone-cre PKM2-KO mouse retinas compared with PKM2-WT retinas (Fig. 9a), suggesting that PKM2 might be expressed more in cones than in rods. Our results also revealed a significant decrease in the gene expression of cone-PDE (PDE6C), medium-wavelength opsin (MWL), short-wavelength opsin (SWL), and cone-transducin-α in cone-cre PKM2-KO mouse retinas compared with PKM2-WT mouse retinas (Fig. 9a). We found no change in the expression of rhodopsin between PKM2-WT and cone-cre PKM2-KO mouse retinas (Fig. 9a). SLC25A33 is a mitochondrial UTP carrier^12^; we found a decreased expression of this protein in cone-cre PKM2-KO mouse retinas compared with PKM2-WT mouse retinas (Fig. 9a), suggesting a possible compromised mitochondrial function in these mice.

**Table 1:** Real-time PCR primers used to measure gene expression

Gene	Forward primer	Reverse primer
HIF-1α	GATGACGGCGACATGGTTTAC	CTCACTGGGCCATTTCTGTGT
GLUT1	TCAACACGGCCTTCACTG	CACGATGCTCAGATAGGACATC
HK-2	GGAACCCAGCTGTTTGACCA	CAGGGGAACGAGAAGGTGAAA
6PGD	AGACAGGCAGCCACTGAGTT	AAGTTCTGGGTTTCGCTCAA
G6PD	CCTACCATCTGGTGGCTGTT	TGGCTTTAAAGAAGGGCTCA
PKM2	ATTGCCCGAGAGGCAGAGGC	ATCAAGGTACAGGCACTACACGCAT
ME1	AGAGGTGTTTGCCCATGAAC	GCTGGTCGGATTACTCAAAGC
ACC1	GGACAGACTGATCGCAGAGAAAG	GCTGTTCCTCAGGCTCACAT
C/EBPβ	GCAAGAGCCGCGACAAG	GGCTCGGGCAGCTGCTT
FAS	GCTGCGGAAACTTCAGGAAAT	AGAGACGTGTCACTCCTGGACTT
PGC1α	GCGCCGTGTGATTTAGCTT	AAAACTCAAAGCGGTCTCTCAA
SREBP-1c	CTGGATTTGGCCCGGGGAGATTC	TGGAGCAGGTGGCGATGAGGTTC
SWL	TGTACATGGTCAACAATCGGA	ACACCATCTCCAGAATGCAAG
MWL	CTCTGCTACCTCCAAGTGTGG	AAGTATAGGGTCCCAGCAGA
RHODOPSIN	CAAGAATCCACTGGGAGATGA	GTGTGTGGGGACAGGAGACT
PDE6C	ACTCCCGAAACTTCAAGTGG	TGGGTGTGATCTCTCCATGA
Cone-Trα	GCATCAGTGCTGAGGACAAA	CTAGGCACTCTTCGGGTGAG
SLC25A33	ATGCAGCTAGAACGCAAGGT	AGCTCCATCTGTGGAGGAGA
18S	TTTGTTGGTTTTCGGAACTGA	CGTTTATGGTCGGAACTACGA

We also used real-time PCR to study whether loss of PKM2 in cones influences fatty acid synthesis and examined fatty acid biosynthesis genes ACC1, C/EBPβ, FAS, PGC1α, and SREBP-1c, using the primers listed in Table 1. We found significantly reduced levels of C/EBPβ, FAS, and SREBP-1c in cone-cre PKM2-KO mice compared with wild-type mice (Fig. 9b). We also examined the expression of genes involved in glycolysis and the pentose phosphate pathway (PPP) in PKM2-WT and cone-cre PKM2-KO mouse retinas via real-time PCR using the primers listed in Table 1. We analyzed the expression of GLUT1, which increases glucose uptake; HK-2 (hexokinase), which phosphorylates glucose; G6PD (glucose 6-phosphate dehydrogenase) and GPGD (6-phosphogluconate dehydrogenase), two enzymes that divert glucose to PPP for NADPH generation; glycolytic genes PKM2 (Fig. 9a) and MEI (malic enzyme 1); and HIF-1α (hypoxia-inducible factor 1α), which regulates the transcription of many glycolytic genes. Our data revealed significantly reduced expression of PKM2, G6PD, 6PGD, GLUT1, and HIF-1α in cone-cre PKM2-KO mouse retinas compared with PKM2-WT mouse retinas (Fig. 9c). We found no significant difference in the expression of HK-2 and ME1 between PKM2-WT and cone-cre PKM2-KO mouse retinas. These findings suggest that there are major alterations in the expression of genes involved in glycolysis and PPP in mice lacking PKM2 in cones.

### Loss of PKM2 in cones results in shortening of cone outer segments

**Fig. 10:**
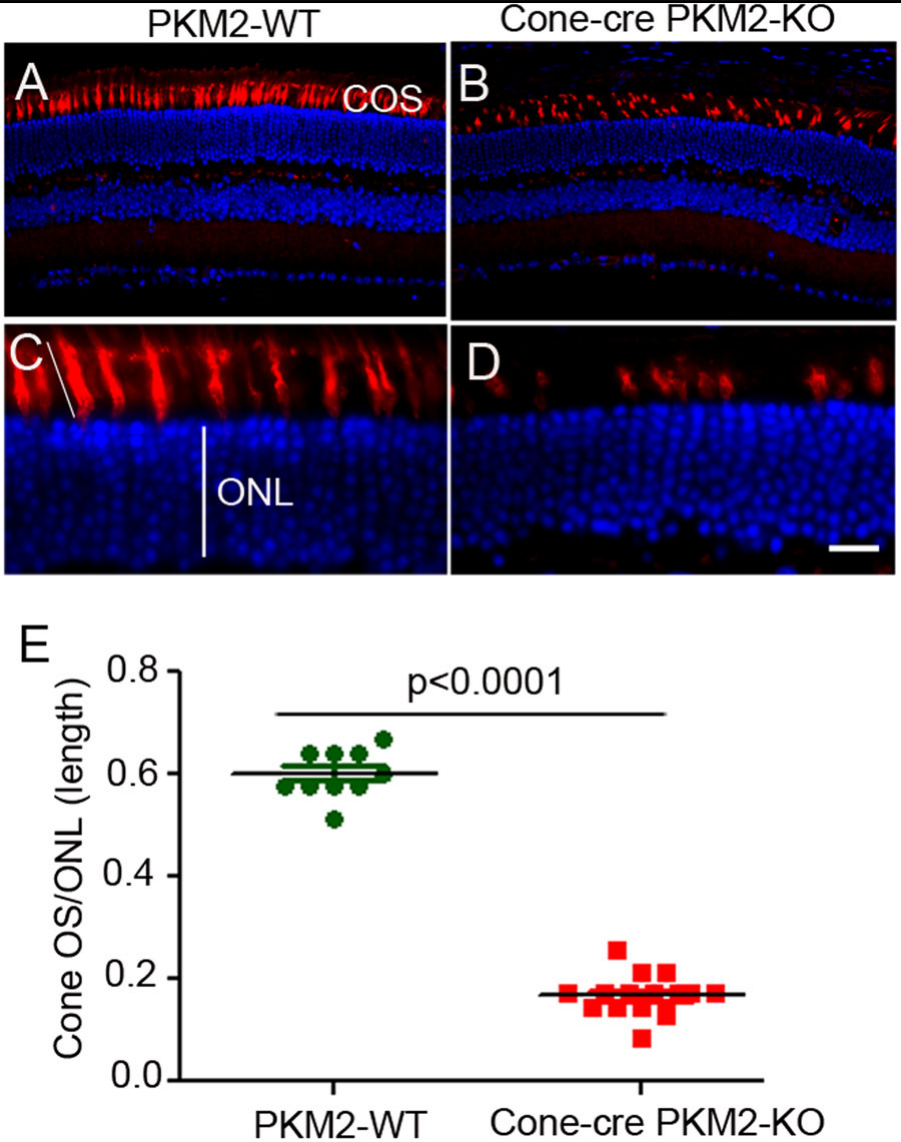
Shortening of cone outer segment length in cone-cre PKM2-KO mice. Prefer-fixed sections of PKM2-WT (**a**) and cone-cre PKM2-KO (**b**) mouse retinas were subjected to immunofluorescence with the anti-PNA antibody. The sections were imaged at ×20 (**a**, **b**), and ×60 (**c**, **d**, scale bar = 20 µm). Using a ruler, the length of cone photoreceptors and outer nuclear layer (ONL) were measured, and the cone outer segment length was normalized to ONL thickness (**e**). Data are mean ± SEM (*n* *=* *16*). Significance was indicated in the panel

Our gene expression studies show a major alteration in gene expression that regulates glucose uptake and phosphorylation, and PPP, the main generator of NADPH generation, as well as genes involved in fatty acid biosynthesis (Fig. 9). We hypothesized that loss of PKM2 in cones may have serious consequences due to alterations in the anabolic processes. Consistent with this hypothesis, we found a significant decrease (70%) in the length of cone photoreceptor outer segments in cones lacking PKM2 compared with cones having PKM2 (Fig. 10). Our results also indicate a reduced length of both S-opsin and M-opsin positive cones in cone-cre PKM2-KO mouse retinas (data not shown). These observations suggest that PKM2 in cones is necessary for cone outer segment maintenance.

## Discussion

In the current study, deletion of PKM2 in cones resulted in structural and functional abnormalities, and the cone degeneration was age-dependent, affecting both S-opsin positive and M-opsin positive cones. The effect of PKM2 loss in cones was more profound on cone function and structure, whereas the PKM2 loss in rods severely affects rod function but has a less severe effect on structure^4^.However, the scotopic b-wave amplitudes in 56-week-old cone-cre PKM2-KO mice were significantly reduced compared to PKM2-WT mice. This scotopic b-wave reduction was surprising because there was no difference in the scotopic a-wave values. We found similar findings previously in aged cone-conditional p85 KO mice^5^. In these mice, we found synaptic changes in the cones that might influence rod b-wave function. We speculate such a possibility cannot be ruled out in cone-conditional PKM2 KO mice.

PKM2 has been thought to cause the accumulation of glycolytic intermediates and increase flux through PPP^2,3^. Studies from other labs have shown that glycolytic reliance promotes anabolism in photoreceptors^8^, and activation of glycolysis promotes photoreceptor survival^13^. In cone-cre PKM2-KO mice, we found decreased expression of genes that regulate glucose uptake and PPP. PKM2 is known to regulate the expression of the glucose transporter GLUT1^14^, and we found reduced gene expression of GLUT1 in cones. Deprivation of glucose from the RPE has been shown to cause the degeneration of cones in animal models of *retinitis pigmentosa* (RP)^15–17^. Thus, the cone cell death in cone-cre PKM2-KO mice could be due to starvation. In cone-cre PKM2-KO mice, PPP-regulated G6PD and 6PGD gene expression are significantly reduced, suggesting decreased NADPH production. We also found a significant reduction in genes involved in fatty acid synthesis in cone-cre PKM2-KO mice, suggesting a defect in fatty acid biosynthesis. We found a highly significant decrease in the expression of fatty acid synthase (FAS) gene in cone-cre PKM2-KO mice compared to PKM2-WT mice. FAS catalyzes the committed step in de novo lipogenesis^18^ and is highly expressed in neural tissue and important for neurogenesis^19^. A recent study showed that deletion of FAS from the neural retina resulted in progressive neurodegeneration and blindness, mimicking an animal model for retinitis pigmenotsa^20^. We^4^ and others^14,21^ have shown PKM2 to be a transcriptional co-activator in gene regulation. We also found that PKM2 enhances FAS promoter activity in vitro (author’s unpublished data). Further studies are required to examine the PKM2 regulated FAS gene expression and de novo lipogenesis in vivo.

In this study, we found that the loss of PKM2 in cones influence the expression of genes involved in glycolysis and fatty acid synthesis in the entire retina. We believe that loss PKM2 in cones somehow influencing other cell types in the retina, such as Muller cells. The reason to believe that fatty acid β-oxidation enzymes are preferentially and predominantly localized to the mitochondria of the Muller cells^22^. Similarly, glycolysis enzymes have also been shown to be localized in Muller cells^23^. It is likely that fatty acid synthesizing enzymes might also be predominantly expressed in the Muller cells. It may be that dying cones in cone-cre PKM2-KO mice could be signaling to Muller cells and that might result in the reduction of gene expression in the retina. Further studies are needed to test this possibility.

Both rod and cone photoreceptors undergo a continuous renewal process,^24^ and there is a high demand for an anabolic process for the synthesis of lipids, protein, and RNA for disc biogenesis. Consistent with these observations, we found shortening of cone outer segments and progressive cone cell death in cone-cre PKM2-KO mice. In both rods^4^ and cones, ablation of PKM2 results in the upregulation of PKM1, and the observed cone degeneration could be due to unfamiliar PKM1 in cone photoreceptors. A recent study reported that in vivo electroporation of a shRNA construct that targets both PKM1 and PKM2 also showed a decrease in OS length, and the decreased OS length was rescued by subretinal injection of PKM2, but not PKM1^8^. These findings show that PKM2 is more important than PKM1 in photoreceptor structure. Our studies clearly suggest that PKM2 is essential for the anabolic process in cones to keep them alive for normal functioning and supports cone structure.

## Materials and Methods

### Antibodies

Polyclonal PKM2 and PKM1 antibodies were obtained from Cell Signaling (Danvers, MA). Polyclonal cone-Trα and goat secondary antibody was procured from Santa Cruz Biotechnology (Santa Cruz, CA). Rabbit polyclonal anti-red/green cone opsin (M-opsin), anti-cone arrestin, anti-S-opsin, and rabbit and mouse secondary antibodies were obtained from Millipore (Billerica, MA). Mouse monoclonal anti-Cre antibody suitable for immunohistochemistry was purchased from Abcam (Cambridge, MA). Monoclonal 1D4 rhodopsin antibody was a kind gift from Dr. James F. McGinnis (University of Oklahoma Health Sciences Center).

### Animals

All animals were treated in accordance with the *ARVO Statement for the Use of Animals in Ophthalmic and Vision Research* and the *NIH Guide for the Care and Use of Laboratory Animals*. The protocols were approved by the Institutional Animal Care and Use Committee at the University of Oklahoma Health Sciences Center. Animals were born and raised in our vivarium and kept under dim cyclic light (40–60 lux, 12 h light/dark cycle). PKM2 floxed mice were purchased from The Jackson Laboratory (Bar Harbor, Maine). We screened wild-type and cone-cre PKM2-KO mice for *rd1* and *rd8* mutations, and all mice were negative for these mutations.

### Chemicals

All reagents were of analytical grade and purchased from Sigma (St. Louis, MO).

### Generation of cone photoreceptor-specific conditional PKM2 knockout mice

Photoreceptor-specific conditional PKM2 knockout mice (cone-cre PKM2-KO) were prepared with the Cre-lox technique by mating floxed PKM2 animals with mice expressing Cre recombinase under the control of the human red/green pigment promoter^5^. In the presence of Cre recombinase, the floxed exon 10 of the *PKM* gene is deleted. The genotype of the rod-cre PKM2 KO mice (i.e., animals carrying the *cre* transgene and homozygous for the PKM2-floxed allele) was confirmed by PCR analysis of tail DNA. For detection of cone-*cre*, we used sense: 5′–GCC GCA TAA CCA GTG AAA CAG CAT–3′ and antisense: 5′–TTG GTT CCC AGC AAA TCC CTC TGA–3′ primers to generate a product size of 500 bp. To distinguish PKM2 floxed allele from wild-type, we used sense: 5′–TAG GGC AGG ACC AAA GGA TTC CCT–3′ and antisense: 5′–CTG GCC CAG AGC CAC TCA CTC TTG–3′ primers to generate 578 bp wild-type allele, 578 bp wild-type, and  680 bp floxed allele in heterozygous, and 680 bp homozygous floxed allele. The wild-type and PKM2 KO mice were on the C57BL/6 background.

### Electroretinography

Flash ERGs were recorded with the Diagnosys Espion E2 ERG system (Diagnosys, LLC, Lowell, MA). Mice were maintained in total darkness overnight and prepared for ERG recording under dim red light. They were anesthetized with ketamine (80–100 mg/kg body weight) and xylazine (5 mg/kg body weight) intramuscularly. One drop of 10% (v/v) phenylephrine was applied to the cornea to dilate the pupil and one drop of 0.5% (v/v) proparacaine HCl was applied for local anesthesia. Mice were kept on a heating pad at 37 °C during recordings. A gold electrode was placed on the cornea, a reference electrode was positioned in the mouth, a ground electrode was placed on the foot, and the mice were placed inside a Ganzfeld illuminating sphere. Responses were differentially amplified, averaged, and stored. For the assessment of rod photoreceptor function (scotopic ERG), we used −3.4, −2.4, −1.4, −0.4, 0.6, 1.6, 2.6 log cd.s/m^2^; to measure cone function (photopic ERG) we used 3.3 log cd.s/m^2^ flash intensity. The amplitude of the a-wave was measured from the pre-stimulus baseline to the a-wave trough. The amplitude of the b-wave was measured from the trough of the a-wave to the peak of the b-wave. For the evaluation of cone function (photopic ERG), a strobe flash stimulus was presented to dilated, light-adapted mice. The amplitude of the cone b-wave was measured from the trough of the a-wave to the peak of the b-wave.

### Preparation of tissue for paraffin sectioning using Prefer as a fixative

Prefer solution (Anatech Ltd, Battle Creek, MI) was used to fix the mouse eyes for 15 min at room temperature, followed by 70% ethanol overnight. The tissue was paraffin-embedded, and 5-μm-thick sections were cut and mounted onto slides. These sections were subjected to immunohistochemistry. A Nikon Eclipse E800 microscope and Olympus MVX10 macroscopic imaging system for high resolution equipped with a digital camera was used to examine the antibody-labeled complexes. Metamorph (Universal Imaging, West Chester, PA) image analysis software was used to capture images under identical microscope and camera settings.

### Immunoblot analysis

Mouse retinas were homogenized in a lysis buffer containing 1% Triton X-100, 137 mM NaCl, 20 mM Tris-HCl (pH 8.0), 10% glycerol, 1 mM EGTA, 1 mM MgCl_2_, 1 mM phenylmethylsulfonyl fluoride, 0.2 mM Na_3_VO_4_, 10 µg/ml leupeptin, and 1 µg/ml aprotinin^25^. Insoluble material was removed by centrifugation at 13,500×*g* for 20 min at 4 °C. The protein concentrations of the solubilized proteins were determined with the bicinchoninic acid reagent following the manufacturer’s instructions (Pierce Biotechnology, Rockford, IL). Five micrograms of retinal proteins were run on 10% SDS-PAGE, followed by protein blotting onto nitrocellulose membranes. After blocking the membranes with 5% non-fat dry milk powder (Bio-Rad) or 5% bovine serum albumin (Sigma) for 45–60 min at room temperature, blots were incubated with anti-rhodopsin (1: 10,000), anti-rod arrestin (1:1000), anti-M-opsin (1:1000), anti-cone arrestin (1:1000), and anti-actin (1:1000) overnight at 4 °C. The blots were then washed and incubated with HRP-coupled anti-mouse, or anti-rabbit secondary antibodies for 60 min at room temperature. After washing, blots were developed with enhanced SuperSignal™ West Dura Extended Duration Substrate (Thermo Fisher Scientific, Waltham, MA) and visualized using a Kodak Imager with chemiluminescence capability. Densitometric analysis of immunoblots was performed in the linear range of detection. Absolute values were then normalized to rod arrestin, and statistical analysis was carried out.

### Statistical analysis

One-way ANOVA and post hoc statistical analysis using Bonferroni’s pairwise comparisons were used to determine statistical significance (*p* *<* *0.05*).

## Electronic supplementary material

Figure S1

Figure S2

Figure S3

Table 1

Figure S4

Supplemental figure legends
