# Supplementary figures and images for: Pyruvate kinase M2 isoform deletion in cone photoreceptors results in age-related cone degeneration
Source: Cell Death Dis. 2018 Jul 3;9(7):737. doi: 10.1038/s41419-018-0712-9 (PMC6030055; doi:10.1038/s41419-018-0712-9)

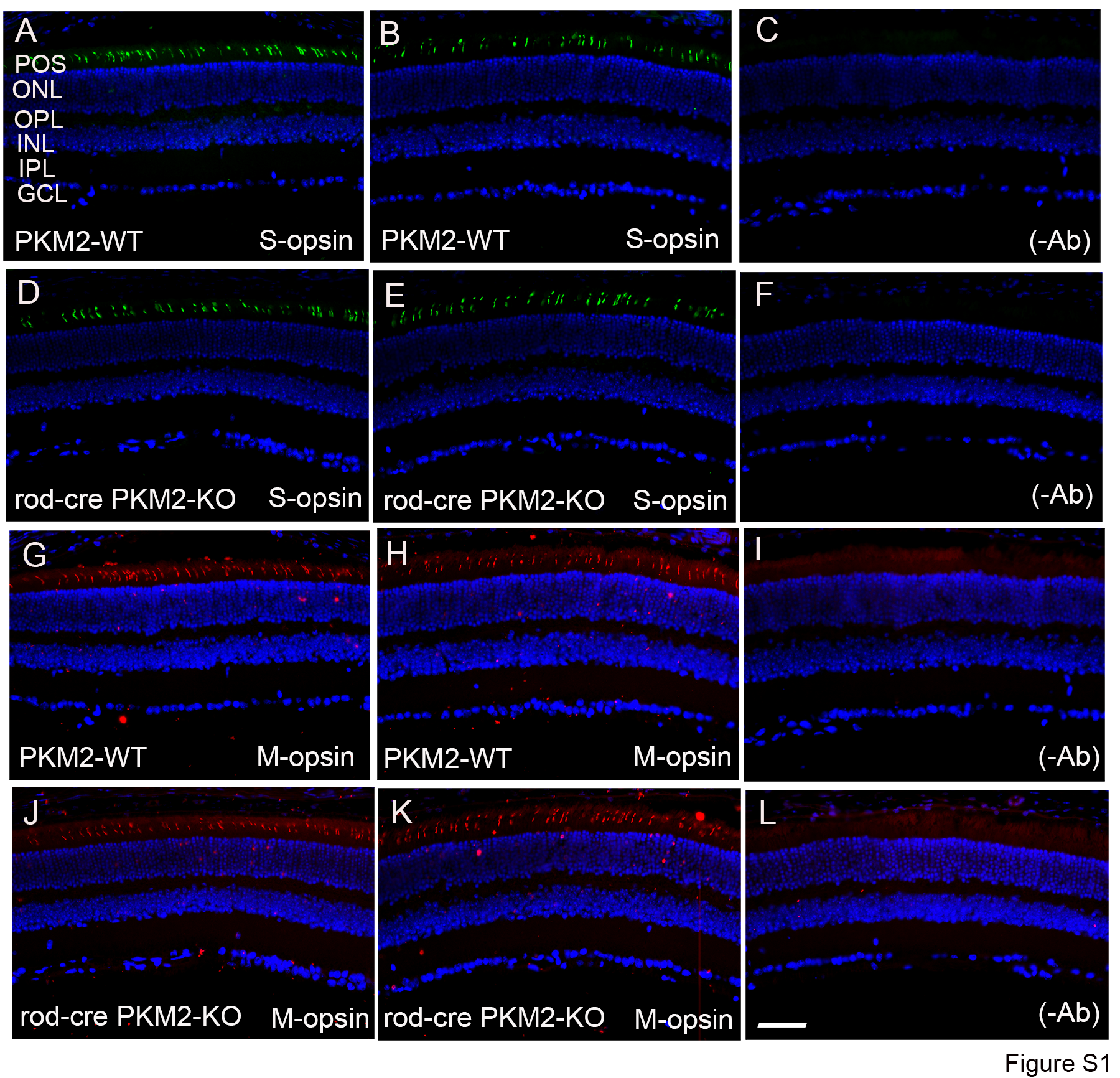

Supplement: Supplementary file 1 — Figure S1 [file 41419_2018_712_MOESM1_ESM.tif]

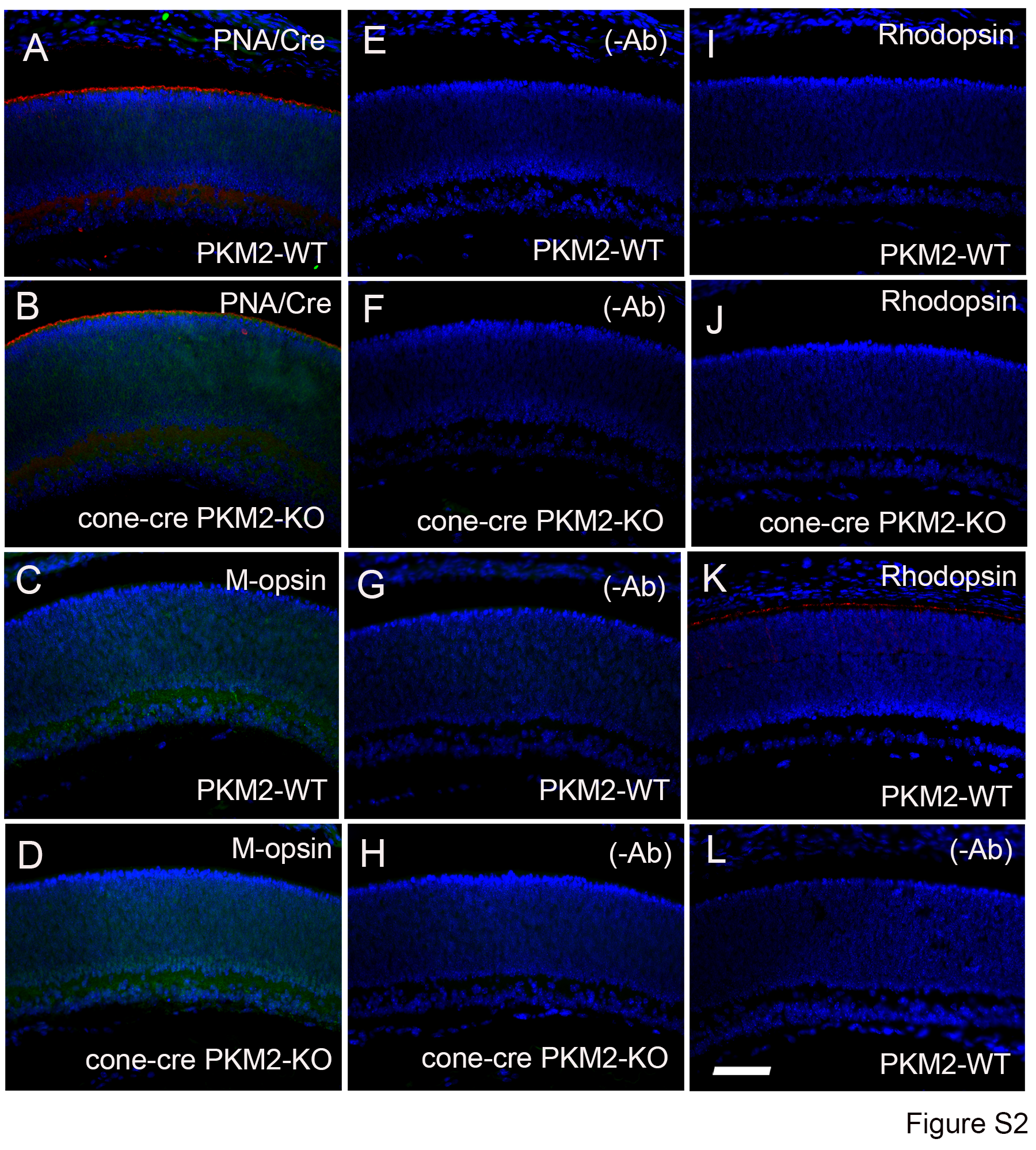

Supplement: Supplementary file 2 — Figure S2 [file 41419_2018_712_MOESM2_ESM.tif]

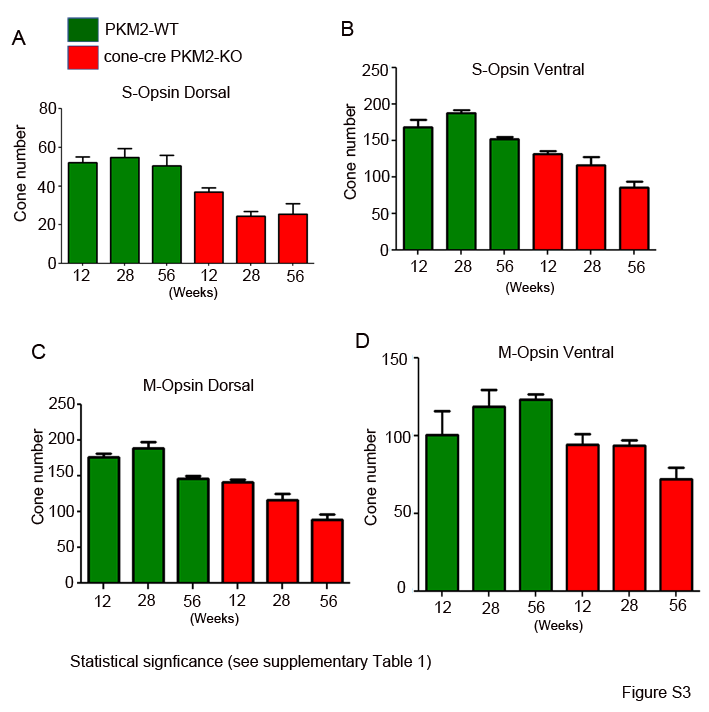

Supplement: Supplementary file 3 — Figure S3 [file 41419_2018_712_MOESM3_ESM.tif]

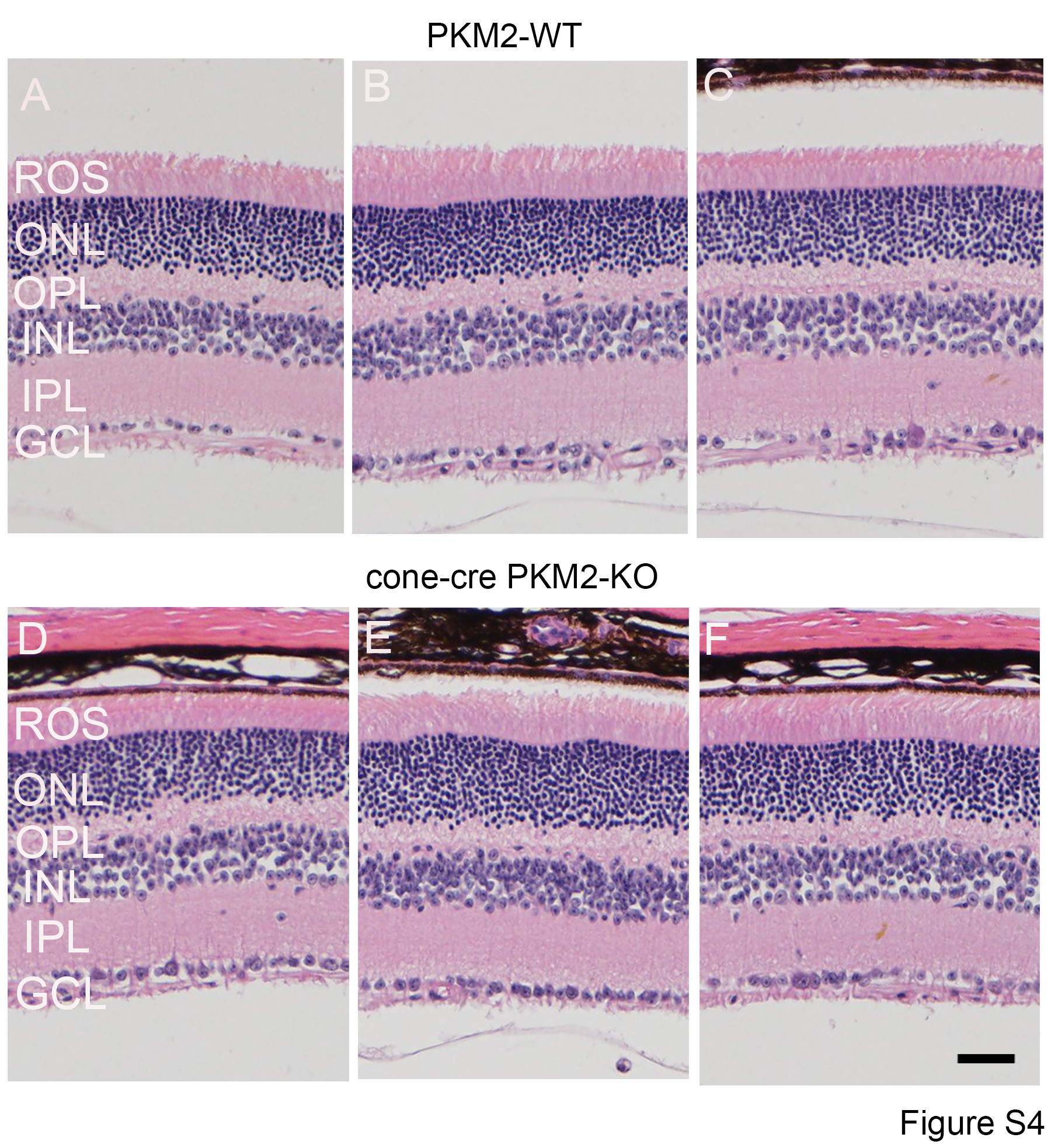

Supplement: Supplementary file 5 — Figure S4 [file 41419_2018_712_MOESM5_ESM.tif]
