# Supplementary material for: Pyruvate kinase M2 isoform deletion in cone photoreceptors results in age-related cone degeneration
Source: Cell Death Dis. 2018 Jul 3;9(7):737. doi: 10.1038/s41419-018-0712-9 (PMC6030055; doi:10.1038/s41419-018-0712-9)
Supplement: Supplementary file 4 — Table 1 [file 41419_2018_712_MOESM4_ESM.docx]

| **S-Opsin Dorsal** | Significance p<0.05 | Summary |
| --- | --- | --- |
| PKM2-KO 20 wk vs PKM2-WT 20 wk | Yes | *** |
| PKM2 KO 20 wk vs PKM2-WT 12 Wk | Yes | ** |
| PKM2-KO 20 wk vs PKM2-WT 56 wk | Yes | *** |
| PKM2 –KO 56 wk vs PKM2-WT 20 wk | Yes | *** |
| PKM2-KO 56 wk vs PKM2-WT 12 wk | Yes | ** |
| PKM2-KO 56 wk vs PKM2-WT 56 wk | Yes | *** |
| PKM2-KO 12 wk vs PKM2-WT-20 2k | Yes | * |
| **S-Opsin Ventral** |  |  |
| PKM2-KO 56 wk vs PKM2-WT 20 wk | Yes | *** |
| PKM2-KO 56 wk vs PKM2-WT 12 wk | Yes | *** |
| PKM2-KO 56 wk vs PKM2-WT 56 wk | Yes | *** |
| PKM2-KO 56 wk vs PKM2-KO 12 wk | Yes | ** |
| PKM2-KO 56 wk vs PKM2-KO 20 wk | Yes | ** |
| PKM2-KO 20 wk vs PKM2-WT 20 wk | Yes | *** |
| PKM2-KO 20 wk vs PKM2-WT 12 wk | Yes | ** |
| PKM2-KO 20 wk vs PKM2-WT 56 wk | Yes | ** |
| PKM2-KO 12 wk vs PKM2-WT 20 wk | Yes | *** |
| PKM2-KO 12 wk vs PKM2-WT 12 wk | Yes | * |
| PKM2-WT 56 wk vs PKM2-WT 20 wk | Yes | ** |
| **M-Opsin Dorsal** |  |  |
| PKM2-KO 56 wk vs PKM2-WT 20 wk | Yes | *** |
| PKM2-KO 56 wk vs PKM2-WT-12 wk | Yes | *** |
| PKM2-KO 56 wk vs PKM2-WT 56 wk | Yes | *** |
| PKM2-WT 56 wk vs PKM2-KO 12 wk | Yes | *** |
| PKM2-WT 56 wk vs PKM2-KO 20 wk | Yes | ** |
| PKM2-KO 20 wk vs PKM2-WT-20 wk | Yes | *** |
| PKM2-WT 20 wk vs PKM2-WT 12 wk | Yes | *** |
| PKM2-KO 20 wk vs PKM2-WT 56 wk | Yes | * |
| PKM2-KO 20 wk vs PKM2 KO 12 wk | Yes | * |
| PKM2-KO 12 wk vs PKM2-WT 20 wk | Yes | *** |
| PKM2-KO 12 wk vs PKM2-WT 12 wk | Yes | ** |
| PKM2-WT 56 wk vs PKM2-WT 20 wk | Yes | *** |
| PKM2-WT 56 wk vs PKM2-WT 20 wk | Yes | * |
| **M-Opsin Ventral** |  |  |
| PKM2-KO 56 wk vs PKM2-WT 56 wk | Yes | ** |
| PKM2-KO 56 wk vs PKM2-WT 20 wk | Yes | ** |

Supplementary Table 1

Significance: * *p*<0.05; ** *p*<0.01; ****p*<0.001.
