# Supplementary material for: Pyruvate kinase M2 isoform deletion in cone photoreceptors results in age-related cone degeneration
Source: Cell Death Dis. 2018 Jul 3;9(7):737. doi: 10.1038/s41419-018-0712-9 (PMC6030055; doi:10.1038/s41419-018-0712-9)
Supplement: Supplementary file 6 — Supplemental figure legends [file 41419_2018_712_MOESM6_ESM.docx]

**Supplementary Figure 1.** **Immunofluorescence analysis of S-opsin and M-opsin in PKM2-WT and rod-cre PKM2-KO mice.** Prefer-fixed sections of PKM2-WT (**A-C**, **G-I)** and rod-cre PKM2-KO (**D-F**, **J-L**) mouse retinas were subjected to immunofluorescence with anti-S-opsin (**A**, **B**, **D**, **E)** and anti-M-opsin (**G**, **H**, **J**, **K**) antibodies. Panels **C**, **F**, **I**, **L** represent the omission of primary antibodies. POS, photoreceptor outer segments; ONL, outer nuclear layer; OPL, outer plexiform layer; INL, inner nuclear layer; IPL, inner plexiform layer; GCL, ganglion cell layer. Scale bar = 50 μm.

**Supplementary Figure 2. Expression of rod photoreceptor markers, cone photoreceptor markers and Cre-recombinase in the developmental retina.** Prefer-fixed sections of P0 (**A-J, L**) and P7 (**K, L**) PKM2-WT (**A, C, E, G, I, K, L**) and P0 cone-cre-PKM2-KO (**B, D, F, H, J**) mouse retinas were subjected to immunofluorescence with anti-PNA (**A, B**), anti-Cre (**A, B**), anti-M-opsin (**C, D**) and anti-rhodopsin (**I, J, K**) antibodies. Panels **A** and **B** represent the merged images of PNA and Cre. Panels **E, F,** **G**, **H,** and **L** represents the omission of primary antibody. ONBL, outer neuroblastic layer; INBL, inner neuroblastic layer. Scale bar = 50 μm.

**Supplementary Figure 3. Age-dependent loss of S-opsin positive and M-opsin positive cones in cone-cre PKM2-KO mice.** Histograms were generated from the PKM2-WT and cone-cre PKM2-KO mice data presented in Figs. 4(**L**), 5 (**M**) and 6 (**M**). The data were plotted as S-opsin dorsal (**A**), S-opsin ventral (**B**), M-opsin dorsal (**C**) and M-opsin ventral (**D**) against each time point (12, 28 and 56 weeks). Data are mean + SEM (*n =6*). One-way ANOVA and post hoc statistical analysis using Bonferroni’s multiple comparisons test were used to determine statistical significance. Statistical significance was presented in Supplementary Table 1.

**Supplementary Figure 4. Morphology of cone-cre PKM2-KO retina and assessment of rod photoreceptor integrity**. Morphologic examination of three independent mouse retinas from 28-week-old PKM2-WT (**A-C**) and cone-cre PKM2-KO mice (**D-F**). ROS, rod outer segments; ONL, outer nuclear layer; OPL, outer plexiform layer; INL, inner nuclear layer; IPL, inner plexiform layer; GCL, ganglion cell layer. Scale bar = 50 μm.
